# *Game-o-Meta*: Trusted Federated Learning Scheme for P2P Gaming Metaverse beyond 5G Networks

**DOI:** 10.3390/s23094201

**Published:** 2023-04-22

**Authors:** Pronaya Bhattacharya, Ashwin Verma, Vivek Kumar Prasad, Sudeep Tanwar, Bharat Bhushan, Bogdan Cristian Florea, Dragos Daniel Taralunga, Fayez Alqahtani, Amr Tolba

**Affiliations:** 1 Department of Computer Science and Engineering, Amity School of Engineering and Technology, and Research and Innovation Cell, Amity University, Kolkata 700135, West Bengal, India; 2 Department of Computer Science and Engineering, Institute of Technology, Nirma University, Ahmedabad 382481, Gujarat, India; 3 Department of Computer Science and Engineering, School of Engineering and Technology, Sharda University, Greater Noida 201310, Uttar Pradesh, India; 4 Department of Applied Electronics and Information Engineering, Faculty of Electronics, Telecommunications and Information Technology, Politehnica University of Bucharest, 061071 Bucharest, Romania; 5 Software Engineering Department, College of Computer and Information Sciences, King Saud University, Riyadh 12372, Saudi Arabia; 6 Computer Science Department, Community College, King Saud University, Riyadh 11437, Saudi Arabia

**Keywords:** P2P gaming, federated learning, metaverse, federated averaging, 5G

## Abstract

The aim of the peer-to-peer (P2P) decentralized gaming industry has shifted towards realistic gaming environment (GE) support for game players (GPs). Recent innovations in the metaverse have motivated the gaming industry to look beyond augmented reality and virtual reality engines, which improve the reality of virtual game worlds. In gaming metaverses (GMs), GPs can play, socialize, and trade virtual objects in the GE. On game servers (GSs), the collected GM data are analyzed by artificial intelligence models to personalize the GE according to the GP. However, communication with GSs suffers from high-end latency, bandwidth concerns, and issues regarding the security and privacy of GP data, which pose a severe threat to the emerging GM landscape. Thus, we proposed a scheme, *Game-o-Meta*, that integrates federated learning in the GE, with GP data being trained on local devices only. We envisioned the GE over a sixth-generation tactile internet service to address the bandwidth and latency issues and assure real-time haptic control. In the GM, the GP’s game tasks are collected and trained on the GS, and then a pre-trained model is downloaded by the GP, which is trained using local data. The proposed scheme was compared against traditional schemes based on parameters such as GP task offloading, GP avatar rendering latency, and GS availability. The results indicated the viability of the proposed scheme.

## 1. Introduction

The modern gaming industry has taken a transformative shift owing to revolutions in computer vision, augmented reality (AR), virtual reality (VR), and 3D-rendering engines [1]. This has changed the entire gaming experience for game players (GPs). Modern games are mostly decentralized and peer-to-peer (P2P)-driven, with peer GPs engaging in gaming environments (GEs), and the data are stored on game servers (GSs) [2]. In VR gaming environments (GEs), GPs play the game (e.g., Beat Saber, Iron Man, or Star Trek) through assisted VR hardware (head-mounted displays and hand controllers), and the GP is immersed in the virtual GE. On the contrary, an AR GE (e.g., Pokemon Go or Sun-seeker) converts the real environment surrounding the GP into a digital interface and overlays new information on the real environment. With the rebranding of Oculus Quest and Facebook renaming itself *Meta*, a giant leap forward is expected in AR/VR GEs, with real-time 360${}^{\circ }$ navigation, object interactions, and haptic device controls having increased four-fold [3]. The next shift will be towards extended reality (XR) engines, enabling the GP’s eye movements and sensory touch (ears, hands, and facial expression) to be rendered and streamed over P2P networks.

**Figure 1:**
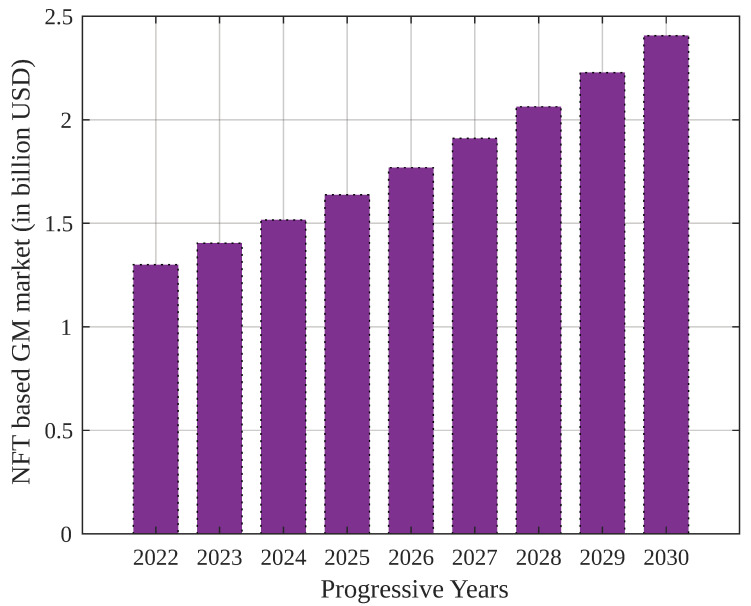
Global market cap of NFT-based gaming metaverses by 2030.

A report by *DappRadar* suggested that the metaverse’s penetration would reach USD 1.3 billion in the gaming industry in 2022 (third quarter). It is estimated that GM projects will drive NFT-based assets and tokens on Web 3.0 platforms, with 912,000 crypto wallets created every day globally. Figure 1 presents the global market cap of NFT-asset-based GMs by 2030, which shows a compounded increase of 8% annually [4].

The increased adoption of metaverses in P2P GEs has propelled researchers to integrate XR-enabled metaverses (networked 3D virtual worlds) in game engines and offer a next-generation gaming experience to GPs. A metaverse creates a virtual environment to emulate real-world objects and human beings by utilizing various IoT-enabled sensors and haptic devices (motion tracking, eye tracking, and voice recognition) with haptic feedback. A metaverse comprises AR/VR systems. A basic VR system needs an accelerometer, a gyroscope, and a magnetometer to create a virtual form of the existing surroundings [5]. AR systems use more sophisticated and complex sensing systems comprising depth sensing; infrared, ambient light, and bio-sensors; and heat-mapping sensors. This enables the AR system to understand the each person’s exact position in the game, what they see and hear, and how it adapts to the game console’s changing environment. Moreover, it helps to create a more immersive and interactive experience for players in the meta environment.

As per the reports by *Statistica* [6], a daily number of 54.1 million concurrent gamers play the metaverse game *Robolox*. The potential metaverse gaming market is expected to reach USD 2.4 billion by 2024. These gaming metaverses (GMs) would offer a holistic XR experience wherein GP avatars can interact, socialize, and earn. For game asset trading, non-fungible tokens (NFTs) could be used as payment mechanisms, and smart contracts (SCs) could be executed to facilitate the asset transfer and selling and purchase of items [7]. Thus, assets would be portable, and transactional details would be stored on the blockchain (BC) [8]. Moreover, GMs could be supported by artificial intelligence (AI)-based engines to personalize and customize the GM environment according to the GP’s likeability [9]. This would improve the Quality-of-Interaction (QoI) of the GPs. The overall experience of playing games can be greatly affected by the QoI. Playing games emphasizing engaging and interacting with players often results in a more enjoyable experience due to QoI. The degree of interactivity a game provides is a critical aspect of the quality of the gameplay experience. Games that provide players with various options, activities, and responses tend to offer a more immersive and captivating experience. Customizable characters and decision making influencing the game’s plot show how games can generate a stronger sense of attachment to the result. The caliber of social interaction among players is also crucial. Multiplayer games that promote collaboration, teamwork, and communication build stronger relationships among players and promote a positive environment. Conversely, games encouraging negative or toxic behavior can damage the social experience and push players to leave. Hence, game developers who prioritize the creation of captivating and immersive experiences, including gameplay and social interaction, are more likely to draw and keep players in the long run.

Game data are normally stored over cloud-assisted game servers (GSs), and AI models are executed to personalize the GE according to the GP. Many confidential GP data (asset information, NFT wallet identifiers, contact addresses, and human sensor data) are stored on GSs for analytics. Thus, any malicious intruder can launch a potential adversarial attack [10]. Secondly, cloud-based GSs suffer from the limitations of huge amounts of traffic, end-user latency, and computational bottlenecks [11].

To address these limitations, federated learning (FL) is a viable choice in GMs and mobile devices. FL has been extensively used for medical ecosystems, as FL utilization in mobile devices enables the support of increasingly complex tasks and operations and contributes to safeguarding user privacy and exploiting the computing resources available on mobile devices [12]. FL is a promising candidate to support the gaming ecosystem due to its high potential. The GE data are trained locally (with the GP’s avatar and personalization data) and are sent to the global model as an update. Coupled with BC, trusted FL training is possible, as local updates are verified as transactional ledgers [13,14].

The remote GPs communicate with the GS over wireless communication infrastructures [15]. Fourth-generation (4G) long-term evolution (LTE) services are preferred. GMs require high-end human–computer interaction (HCI) support, 3D modeling, and edge computing and caching support [16,17]. A recent study by Cheng et al. [18] suggested fifth-generation (5G) network services for improved coverage and caching. The supported throughput in 5G networks is ≈10−20 Gbps, and to improve HCI, tactile internet (TI) channels are preferred to support dynamic XR content. However, in the near future, the large number of concurrent GP connections could require a shift towards sixth-generation (6G) services. By 2030, it is predicted that there will be a need for 6G networks to support up to 125 billion wireless devices [19]. To enable this, creating a smart signal and data-processing system that enables distributed learning is essential. FL is a critical technology that has the potential to fulfill the expected requirements of 6G networks [20]. 6G-TI is envisioned to support a low packet loss rate in the order of $10^{-9}$, with a 0.1 ms end-to-end delay, which is suitable for real-time haptic interactions. 6G-TI operates on the THz band, with a low outage probability of $10^{-7}$ [21]. Thus, 6G-TI integration with GMs would improve the GP QoI, as real-time rendering and control would be possible.

Motivated by the above discussion, we present an integrative scheme, named *Game-o-Meta*, which combines FL in a 6G-P2P GM environment. The scheme ensures responsive control for GPs (the avatars in the GM), and the communication is handled with a low latency and delay. FL ensures the customization of the GE for specific GPs based on local data training. Offline storage via interplanetary file systems (IPFSs) is used for the GP data, and the meta records are stored on the BC to ensure trust between heterogeneous P2P game servers. In our scheme, we considered an optimized version of the federated averaging (FedAvg) algorithm. We proposed a parallel version of the FedAvg algorithm, named parallel FedAvg (P-FedAvg), that overcomes the drawbacks of the traditional FedAvg scheme, where a centralized parameter server is responsible for communication with all local FL clients. The traditional FedAvg algorithm suffers from computational bottlenecks in high-load conditions and would significantly affect the learning rate. The reason for this is trivial, as clients might be located at heterogeneous sites, and it is not easy to establish a network with high availability across all links. Our approach divided the entire network into small regions controlled by its regional parameter server.

The research contributions of this article can be summarized as follows.

- A framework for GP interaction in a 6G-P2P decentralized GM environment is proposed, whereby the GPs can socialize and interact.
- Further, a model of the interaction between two game clients is presented, where the clients form game avatars and interact in the GE through an XR-enabled system. The clients can perform transactions of assets via NFT tokens, and the game state is stored in the local IPFS server.
- The article presents a BC-based gradient-exchange model and a threat and privacy model, followed by the problem formulation.
- Based on the problem, an optimized parallel federated averaging (P-FedAvg) algorithm is presented, which trains the local data and GE parameters collected from the GM and computes the local gradients to fine-tune the global model hyperparameters. The global model state is stored in a game chain, and game clients download the updated parameters.
- The performance of the aforementioned proposed scheme was validated using the parameters of the mining cost, rendering latency, and energy consumption.

The rest of the article is organized as follows. Section 2 discusses the entities, the working flow, and the problem formulation of the proposed scheme. Section 3 presents the interaction model and the FL learning algorithm based on the problem formulation. Section 4 presents the performance analysis of the scheme, and, finally, Section 5 concludes the article.

### 1.1. Related Work

This section presents the existing GM, FL, and AR/VR ecosystem schemes. Researchers have been inspired to incorporate metaverses (networked 3D virtual worlds) into game engines and provide GPs with a cutting-edge gaming experience, resulting in the rising acceptance of XR in P2P GEs. This section discusses various articles related to gaming scenarios, metaverses, and FL model training scenarios.

Regarding FL-based mechanisms, Zou et al. [22] presented a scheme that integrated FL and game theory for a dynamic strategy and the rationing of optimal participants in distributed learning ecosystems. The accuracy and consumption of energy were identified using cost metrics. For decentralized environments, a mobile crowd FL approach was presented. The scheme ensured profit maximization via training rewards and cost metrics over associated distributed servers and devices. These metrics were deployed using two strategies: accuracy- and size-based policies. Sun et al. [23] combined FL with mobile augmented reality in a metaverse realm. The aim was to perform resource-intensive network operations in distributed systems at an extremely low latency in order to induce a quick response from avatars. The gaming systems had financial transaction modes supported through an NFT and cryptocurrency exchange platform. Park et al. [24] presented a case study on channel access mechanisms for both orthogonal and non-orthogonal policies in the metaverse. The authors presented the concept of semantic communication in the metaverse, which was used for real-time interactions among diverse entities. The authors called this the semantic metaverse (SM). In the SM, the details of the gaming participants and the environmental conditions were analyzed based on reinforcement learning methodologies. The learning models formed an optimal-fit policy to bundle the events and actions together for optimal QoI among the GPs. The scheme proposed a 6G communication channel on the wireless communication front.

Regarding the networking aspects of FL models in the metaverse, Latif et al. [25] discussed the emerging wireless key performance indicators (KPIs) and their role in avatar generation, rendering, twin modeling, and deployment in the metaverse. The paper discussed twin signaling, twin applications, the scheduling of resources through radio signals, proactive traffic analysis, traffic predictions, and intelligent media control rates for optimal network utilization. The overall space was divided into physical and meta-space, and wireless interfaces carried out interactions. Networking metrics for QoS were presented, such as reliability, latency, and a low error rate. In addition to this, security mechanisms for human-centric and emotion-driven model data transfer were also presented. A potential limitation of increased mobile usage was presented, which accounted for the increased complexity of data handling in the metaverse. To solve this limitation, Lin et al. [26] presented the concept of sparse samples, which also dealt with the associated security issues, and proposed an algorithm called federated multi-task inverse soft actor-critic. Here, the roles of the actors and their avatars were given importance in relation to the connected environment. Zelenyanszki et al. [27] considered the privacy implications of NFT avatars. NFTs are one-of-a-kind tokens that can symbolize anything from art to audio to a digital/virtual user participating in the distributed virtual environment. Zhou et al. [28] focused on an FL-assisted mobile AR (MAR) system using non-orthogonal multiple access (NOMA) and proposed an algorithm to optimize time, energy, and model accuracy simultaneously.

Secured and trusted metaverse environments have recently been proposed. For example, Hui et al. [29] assessed the quality of the aggregated model maximization problem and proposed the MAXQ high-quality function to achieve a better decision-making process. Zhang et al. [30] proposed a differential privacy scheme that was used for interactions between participants in a decentralized scenario. A game-theoretic concept that supported budget-balanced, truthful, and individually rational mechanisms was presented for transactional procedures. An influence reversal mechanism, GAMES, in an FL-based environment with an ML framework was discussed in Gupta et al. [31]. The client heterogeneity under distribution alterations in FL was examined structurally. Specifically, the FL GAMES model for learning stable causal representations across clients was designed and tested Lee et al. [32] proposed an asynchronous FL process and analyzed the convergence. Additionally, transmission scheduling in a wireless distributed learning network was explored to enhance the learning process in the distributed ecosystem. Fan et al. [33] proposed a BC-based incentive mechanism for a trading platform and secured transfer via a game-theoretic approach that eventually presented an optimal Nash equilibrium. Regarding FL-based incentive and resource optimization, Jiang et al. [34] proposed a scheme based on the Stackelberg equilibrium for resource optimization and incentive preservation. Yu et al. [35] proposed an FL-incentivized payoff-sharing scheme to incentivize FL data owners to contribute high-quality data to the data federation. This approach also took account of the various factors critical to FL while distinguishing between concerns related to the delay in the federated model’s revenue-generation scheme. Table 1 presents a comparative analysis of the proposed sheme and existing state-of-the-art (SOTA) approaches.

### 1.2. Research Gap

As discussed in Section 1.1, most recent studies on metaverses for gaming have focused on avatar formation, metaverse engines, and the details of the game design. However, to realize an end-to-end solution, it is imperative to design a scheme for GMs wherein the game data are analyzed and the GPs’ privacy is preserved (including players’ wearable devices and haptic feedback). The sensor data are highly sensitive, because they include a player’s physical movements, motion capture, and physiological and environmental monitoring data. In addition, some metaverse games also collect players’ personal information, such as biometric data (i.e., voice and facial recognition); location; and behavior patterns. Furthermore, metaverse GEs are often accessed via an online interface (public internet), which implies that the sensing data collected by GEs are transmitted over the internet. Thus, if these data are not properly managed (i.e., encrypted), they could be easily intercepted by attackers, potentially leading to a data breach or data theft attack. If adversaries access these data, they can be used for identity theft, fraud, and other nefarious activities. Therefore, a proactive mechanism is required to tackle security- and privacy-related issues associated with metaverse GEs.

Very few studies have focused on integrating FL with metaverses, and, to the best of our knowledge, this is the first scheme to combine the metaverse and FL for GEs. Further, a GM requires quick interactions and control; thus, we present the scheme against the backdrop of the 6G-TI channel, and we utilized enhanced mobile broadband (FeMBB) for bandwidth management. For transactional control, we present an NFT engine in a GM that allows the trading of items among GP avatars. Recent studies have noted that, due to the increase in the use of mobile devices and other ubiquitous computing equipment, a growth in the utilities of metaverses has been observed. The proposed scheme involves a decentralized gaming scenario. The players create the avatars using the metaverse for a real-time experience. These interactions between avatars must be sufficiently secured and fast. Hence, supporting technologies such as 6G, NFTs, and ERCs and connectivity between the local storage and file-sharing systems such as IPFSs with the integration of FL were included.

## 2. *Game-o-Meta*: Problem Formulation

**Figure 2:**
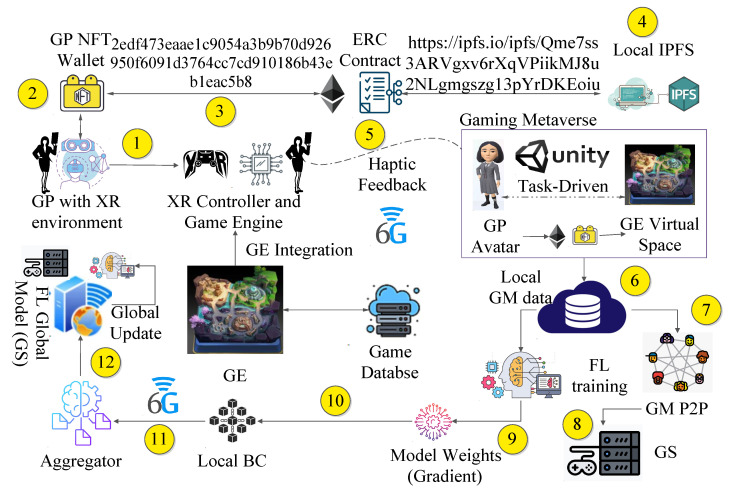
*Game-o-Meta*: The proposed flow model.

This section presents the entities and flow of the proposed scheme. Figure 2 presents the GM environment and the overall GP interaction with the metaverse.

Initially, we present the scheme’s entities and proceed with the problem formulation.

- *Game players*: We considered *n* GPs, represented as $\left\{GP_{1},GP_{2},\ldots ,GP_{n}\right\}$. Every GP has an XR-enabled headset and controller $\left\{H_{1},H_{2},\ldots ,H_{n}\right\}$, which allows the GP to interact in the GM. A trivial assumption for a P2P environment requires all GPs to be globally distributed. The XR controllers allow the players to interact with *p* games, represented by $\left\{GO_{1},GO_{2},\ldots ,GO_{p}\right\}$.
- *Game avatars*: Once any $GP_{n}$ registers to a specific game $GO_{m}$, a mapping ID is created M:GP→GO, which is denoted as GID(n,p). Based on GID(n,p), we constructed a metaverse environment wherein a game avatar GA was created. The GM includes the following details of the GP:
  (1) $$\begin{array}{c} GA=\{MID\left(n,p\right),V_{is},W_{GP},A_{GP}\} \end{array}$$
  where MID(n,p) denotes the metaverse ID of user *n* mapped to game *p*, $V_{is}$ is the GE virtual space, $W_{GP}$ is the crypto wallet of GP, and $A_{GP}$ is the NFT assets (tokens) of the GP in the GM ecosystem.

### 2.1. The Scheme Flow

1. Any $GP_{n}$ interacts with the XR environment via a controller $XR_{C}$.
2. $GP_{n}$ sets up its NFT wallet and decides the assets $A_{GP}$ it can trade in the GM ecospace.
3. Once $A_{GP}$ is fixed, the information is registered in a smart contract via the ERC 721 token.
4. The details of the contract and the $A_{GP}$ are stored in a local IPFS, which is accessible to the user via a 32-byte IPFS content key, represented as $IPFS(GP_{n})$.
5. GP selects the game engine and the XR environment $XR_{Env}$ on $XR_{C}$. A virtual space is created via the GE, and the GP selects a local avatar, $GO_{n}$, to represent itself in the GM. The haptic control and communication from the GP to the GM is managed through a 6G-TI channel.
6. The local GM data are stored in a shared metaverse database, which is encrypted by two sets of keys, $\left\{K_{mpub},K_{gpr}\right\}$, where $K_{mpub}$ represents the accessible public GM key, and $K_{gpr}$ is the private key of the GP.
7. Based on the local data, a local P2P server is connected as a game server (GS), and the P2P game starts with other GPs (via their GO form in the GM).
8. An FL training process is initiated by the GS, which ensures the privacy of the GP data and customizes the game according to player interactions, which improves the gamer’s experience.
9. Based on the game duration and GM interactions via GO in the GM, the local FL model weights are updated.
10. The updated FL model weights *w* are stored in a local BC.
11. The weights are communicated to the mobile aggregator node *M*.
12. The aggregator finally updates the global server model, which shares the updated weights and parameters with the local FL models.

### 2.2. Network Model

A 6G-TI channel *C* was considered, wherein the bandwidth was managed among *n* GPs, and the allocation was managed through a network function virtualization (NFV) operator *O*, which operated over the FeMBB service. As there were *n* GPs, we considered the link bandwidth split ratio *R*, where every user received a share of C/R. At any given time τ, the individual bandwidth function is denoted as follows:

(2) $$\begin{array}{c} B_{n}\left(\tau \right)=\delta_{n}\left(\tau \right)C_{n} \end{array}$$

where $B_{n}\left(\tau \right)$ denotes the bandwidth allocated to GP *n*, $\delta_{n}\left(\tau \right)$ denotes the allocated capacity at time τ, and $C_{n}$ denotes the allocation NFV constant. Based on this, the traffic demand $T_{d}$ cannot exceed the overall allocation, denoted as follows:

(3) $$\begin{array}{c} T_{d}\leq \sum_{i=1}^{n}B_{n}\left(\tau \right) \end{array}$$

### 2.3. Transaction Model

In $V_{is}$, we considered any two GPs, denoted by $GP_{a}$ and $GP_{b}$, interacting via their wallets $WP_{a}$ and $WP_{b}$. The wallets are linked to an Ethereum contract *C*, and virtual NFT tokens $N_{a}$ and $N_{b}$ are generated for asset transfer. The contract contains the following details:

(4) $$\begin{array}{c} SC=\{MID\left(n,p\right),WP_{a},WP_{b},N_{a},N_{b},A,P\} \end{array}$$

where *A* denotes the asset ID for transfer, and *P* denotes the asset price. A local contract address is used to execute the ownership transfer in the GM, stored in the local IPFS of users *A* and *B*. The IPFS receives the previous weights $p_{n}^{t}$, overall set of mobile devices $D_{n}$, and local data *D*.

(5) $$\begin{array}{c} ID\leftarrow IPFS(D,D_{n},Dp_{n}^{t}) \end{array}$$

Once the 32-bit content address is generated, it is added as the data part of the transaction. The block also contains version *V*, the hash of the previous block $H_{p}$, the Merkle root $M_{r}$, the time stamp *T*, and the nonce value *N* [36].

(6) $$\begin{array}{c} Bk\leftarrow (V,H_{p},M_{r},T,N,ID) \end{array}$$

For gradient exchange, we used BC, whereby the local model updates are exchanged. We assumed that each GP generates a gradient update denoted by $g_{a}$ and $g_{b}$ for their respective local models. The gradients are then encrypted using homomorphic encryption before being sent to the BC network.

Let $HE_{k}$ be the homomorphic encryption function with the public key *k*. The encrypted gradients are denoted by $HE_{k}\left(g_{a}\right)$ and $HE_{k}\left(g_{b}\right)$. The BC stores the encrypted gradients along with their corresponding transaction IDs. The process of gradient exchange in BC can be defined as follows:

1. $GP_{a}$ generates the gradient update $g_{a}$ for its local model.
2. $g_{a}$ is encrypted using homomorphic encryption, denoted as $HE_{k}\left(g_{a}\right)$.
3. The encrypted gradient $HE_{k}\left(g_{a}\right)$ is added to a transaction $Tx_{a}$ along with its metadata and is broadcast to the network.
4. $GP_{b}$ receives the transaction $Tx_{a}$ and decrypts the gradient using its private key: $g_{a}=DE_{k}\left(HE_{k}\left(g_{a}\right)\right)$.
5. $GP_{b}$ generates its gradient update $g_{b}$ for its local model.
6. $g_{b}$ is encrypted using homomorphic encryption, denoted by $HE_{k}\left(g_{b}\right)$.
7. The encrypted gradient $HE_{k}\left(g_{b}\right)$ is added to a transaction $Tx_{b}$ along with its metadata and is broadcast to the network.
8. $GP_{a}$ receives the transaction $Tx_{b}$ and decrypts the gradient using its private key: $g_{b}=DE_{k}\left(HE_{k}\left(g_{b}\right)\right)$.
9. The decrypted gradients $g_{a}$ and $g_{b}$ are then used to update the global model on the game server.

The use of homomorphic encryption ensures the privacy of the gradients during the exchange process, as only the authorized GPs can decrypt the gradients.

### 2.4. FL Model

Two core operations were considered for FL: the local updates stored in the GM-P2P server and the weight aggregation. For any general iteration *t*, we considered that mobile device $D_{n}$ updates the model weights as follows:

(7) $$\begin{array}{c} p_{n}^{t+1}=p_{n}^{t}-\eta \Delta l\left(p_{n}^{t}\right) \end{array}$$

where $p_{n}^{t}$ denotes the previous weights from the device $D_{m}$, η denotes the learning rate of the model, and Δl denotes the loss function. The weights are updated based on the FedAvg algorithm, represented as follows:

(8) $$\begin{array}{c} w_{0}^{t+1}=\sum_{n\in D_{n}}\frac{D_{n}}{D}p_{n}^{t} \end{array}$$

where $D_{n}$ denotes the overall set of mobile devices, and $w_{0}^{t+1}$ denotes the averaged weight of the learning model after the $t^{th}$ iteration. $D_{n}$ shows the size of individual $GP_{n}$’s data in the GS, and *D* is the overall local data size. More specifically, $\frac{D_{n}}{D}$ denotes the importance (contribution) of a GP in the learning process.

### 2.5. Threat Model

We considered a threat model with three types of attackers: passive eavesdroppers, active attackers, and compromised peers. Let us denote the set of all peer devices as *R*, the set of compromised peers as $C_{R}$, and the set of honest peers as $H=R/\;C_{R}$. We also denote the set of all GSs as *S*. The attacks are presented below.

*Passive eavesdropping*: Passive eavesdroppers $A_{pe}$ aim to intercept and read the communication between peers and GSs without modifying the data. We assumed that passive eavesdroppers can intercept all network traffic, including encrypted gradients and weights, and have the ability to perform traffic analysis to infer information about the training data and model. The passive eavesdroppers’ attacks can be represented as follows:

(9) $$\begin{array}{c} A_{pe}=\left\{\mathrm{intercept}\;\mathrm{all}\;\mathrm{network}\;\mathrm{traffic}\right\} \end{array}$$

*Active attackers*: Active attackers $A_{aa}$ aim to modify the data being exchanged between peers and game servers to manipulate the FL process. We assumed that active attackers can modify network traffic in transit, including encrypted gradients and weights, and perform man-in-the-middle attacks. The active attackers’ attacks can be represented as follows:

(10) $$\begin{array}{c} A_{aa}=\left\{\mathrm{modify}\;\mathrm{network}\;\mathrm{traffic}\;\mathrm{in}\;\mathrm{transit}\right\} \end{array}$$

*Compromised peers*: Compromised peers $A_{cp}$ aim to manipulate the FL process by sending incorrect gradients or weights. We assumed that an external attacker controls compromised peers and can perform arbitrary computations. The compromised peers’ attacks can be represented as follows:

(11) $$\begin{array}{c} A_{cp}=\left\{\mathrm{send}\;\mathrm{incorrect}\;\mathrm{gradients}\;\mathrm{or}\;\mathrm{weights}\right\} \end{array}$$

Thus, the overall threat model is presented as follows:

(12) $$\begin{array}{c} T=\{R,C_{R},A_{pe},A_{aa},A_{cp}\} \end{array}$$

### 2.6. Differential Privacy Model

Based on the threat model, we needed to formulate a privacy-preservation FL scheme for GPs in the GM. Let *x* be the private information of a GP and *y* be the noisy FL weights uploaded by the GP to the BC. The privacy guarantee of the proposed mechanism was defined in terms of the differential privacy parameter *c* and the privacy budget ϵ.

We assumed that the noise added to the FL weights satisfied the following conditions:

1. The noise is independent and identically distributed (i.i.d.) and Laplace distributed with a mean of 0 and a scale parameter *c*.
2. The differential privacy parameter *c* satisfies the following condition:
  (13) $$\begin{array}{c} c\geq \frac{\ln(1/\delta )}{\epsilon } \end{array}$$
  where δ is the probability of the failure of the differential privacy mechanism, and ϵ is the privacy budget.

Under these constraints, we could prove that the proposed mechanism provided a differential privacy guarantee by analyzing the impact of the noise on the FL weights. Let H(x) and H(y) be the histograms of the private information *x* and the noisy FL weights *y*, respectively. The privacy guarantee can be expressed by the relative entropy between the histograms H(x) and H(y), as follows:

(14) $$\begin{array}{c} D(H(x)||H(y))\leq \epsilon \end{array}$$

where *D* is the relative entropy function. Using the properties of the Laplace distribution and the assumption that the noise is i.i.d. and Laplace distributed, we showed that the relative entropy could be upper-bounded as follows:

(15) $$\begin{array}{c} D\left(H\left(x\right)||H\left(y\right)\right)\leq {c||x||}_{1} \end{array}$$

where ${\left||x|\right|}_{1}$ is the L1 norm of the private information *x*. Therefore, the privacy guarantee could be rewritten as follows:

(16) $$\begin{array}{c} {c||x||}_{1}\leq \epsilon \end{array}$$

which implies that

(17) $$\begin{array}{c} {\left||x|\right|}_{1}\leq \frac{\epsilon }{c} \end{array}$$

Thus, by appropriately setting the value of the differential privacy parameter *c*, we could control the amount of privacy protection the mechanism provides. Thus, the proposed mechanism effectively protects the privacy of GPs in the metaverse by adding differential noise to the FL weights. The model provides a privacy guarantee regarding the differential privacy parameter and the privacy budget and can be tuned to balance privacy and utility. Based on the above discussion, we present the problem objectives below.

- *Maximize $B_{n}\left(\tau \right)$*: We intended to maximize the individual GP bandwidth to maximize the overall user experience.
- *Minimize SC latency l*: When $GP_{a}$ and $GP_{b}$ interacted in the GM, the asset transfer latency needed to be minimal via gameplay.
- *Maximize the accuracy of $\left(p_{n}^{t}\right)$*: At any given iteration *t*, the accuracy of the FL model needed to be maximized.

Thus, the problem was considered convex based on the abovementioned objectives and hence needed to be broken into sub-optimal problem sets SO(l).

(18) $$\begin{array}{c} F\left(B_{n}\left(\tau \right),SC_{l},p_{n}^{t}\right)=max\left(SO\left(l_{1}\right),-SO\left(l_{2}\right),SO\left(l_{3}\right)\right) \end{array}$$

## 3. *Game-o-Meta*: The Proposed Scheme

**Figure 3:**
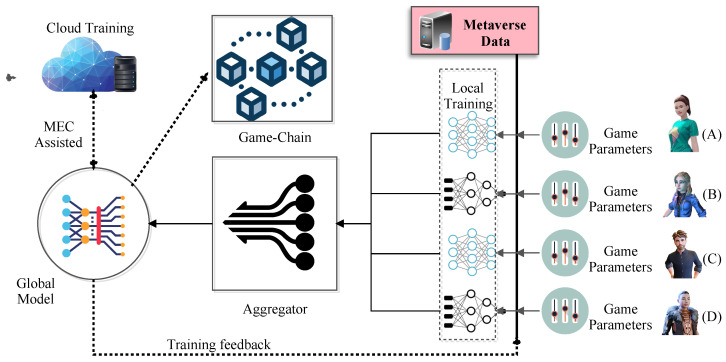
*Game-o-Meta*: The global parallel FedAvg model.

In this section, based on Equation (18), we present a parallel optimization of the FedAvg scheme (P-FedAvg), wherein we considered that the global model GMod was distributed into *q* different parallel server models, $\left\{MP_{1},MP_{2},\ldots ,MP_{q}\right\}$. Figure 3 presents the details of the global aggregation model, wherein the game parameters from different GPs (denoted by players A, B, C, and D in the figure) are fed into a subset of $G_{mod}$. To present the same, we considered any $i^{th}$ MP as a subset of the total GPs connected to $MP_{i}$. A trivial condition was followed, whereby the assignment of GP to $MP_{i}$ and $MP_{j}$ were distinct, and thus $GP_{MP_{i}}\cap GP_{MP_{j}}=\phi$.

Any $MP_{i}$ and $MP_{j}$ communicate by forming a ring topology, whereby each MP is connected to its two nearest neighbors in terms of network connectivity and not based on physical location. This assumption is trivial, as the nearest MP neighbor might be dynamic, and thus the closest MP connected through a network link (protocol) would be considered a neighbor. Thus, all these MPs are connected circularly. The topology implementation is carried out by defining an array of size MAX, where MAX denotes the total number of MPs in the system. Each element of the array corresponds to an MP in the ring. The index of the array represents the identifier (ID) of the MP.

The following process could help in determining the neighbors of an MP with the ID *i*. The left neighbor is assigned ID (i−1), and the ID would wrap around if i=0. The right neighbor is given ID (i+1), and the ID would wrap to 0 if i=(MAX−1), where max is the maximum size of the array. In this way, we could easily determine the neighbor of a given MP in the ring based on the ID. Any node sends a message to its left or right neighbors, and the message is passed through the ring until it reaches the destination MP.

Based on the ring topology assumption, we considered that $MP_{i}$ and $MP_{j}$ communicate with each other to share local transactional data, forming a matrix $M_{m\times m}$. The entry is $M_{ij}=1$ if the PS has communicated; otherwise, it is $M_{ij}=0$.

For any $GP_{i}$, we considered the local data sample as $D_{i}$, and, similarly, as $D_{j}$ for any $GP_{j}$. The objective was to train the global $MP_{i}$ to which both $GP_{i}$ and $GP_{j}$ are assigned. For the training problem, we considered the reinforcement FL (RFL) scheme, and the local $D_{i}$ at time *t* for any GP is presented as $D_{i,t}$. We could represent the optimization problem for the $i^{th}$ GP at time *t* as follows:

(19) $$\begin{array}{c} \min_{w\in R^{d}}F_{i}\left(w\right)=E_{\left(x,y\right)∼D_{i,t}}\left[\ell (w,(x,y))\right]+\lambda R\left(w\right) \end{array}$$

where *w* is the model parameters, *ℓ* is the loss function, *R* is the regularizer, and λ is the regularization strength. The equation could be modified as follows:

(20) $$\begin{array}{c} \min_{w\in R^{d}}F_{i}\left(w\right)=E_{\left(x,y,r\right)∼D_{i,t}}\left[\ell \left(w,\left(x,y\right)\right)+\gamma rV_{i-1}\left(x^{\prime }\right)\right]+\lambda R\left(w\right), \end{array}$$

where γ is the discount factor, *r* is the reward signal, $V_{i-1}\left(x^{\prime }\right)$ is the value function at the previous time step, and $x^{\prime }$ is the next state. We could use Q-learning to estimate the value function as follows:

(21) $$\begin{array}{c} V_{i-1}\left(x\right)=\max_{a}Q_{i-1}\left(x,a\right), \end{array}$$

where *a* is the action, and $Q_{i-1}\left(x,a\right)$ is the Q-value function at the previous time step. We used the P-FedAvg algorithm to optimize the above equation in a parallel and distributed manner, so that the $i^{th}$ GP communicates with the $j^{th}$ GP to share local transactional data, and the global model is distributed into *q* different parallel server models.

In P-FedAvg, to maximize $p_{n}^{t}$, we needed to minimize the loss function L(f), computed as follows:

(22) $$\begin{array}{c} L\left(f\right)=\sum_{i=1}^{S}1/S_{i}\sum_{i\in N_{s}}\frac{1}{N_{s}}f_{j}\left(x\right) \end{array}$$

where *S* denotes the cardinality of the GP assigned to any $MP_{i}$, and $N_{s}$ denotes the load size. The parallel FedAvg algorithm proceeds as follows:

- Every $MP_{i}$ initially distributes the global model parameters obtained from GMod to its sub-global model MP to start the initial round.
- Based on local weights, the MP optimizes the parameter value and passes it to the client batch assigned under it.
- Each $GP_{i}$ runs *t* rounds of local model iteration and updates the model weights, which are returned to its local $MP_{i}$.
- GMod sets up a local value of the loss function, which it optimizes at each response round from $MP_{i}$. The process terminates once the loss converges to the optimal value.

Every $GP_{i}$ initiates a parameter vector, denoted by $G_{i}$, which requires intermediate vector $\gamma_{i}$ (represented as $\frac{1}{K_{i}}\sum_{i\in K_{i}}G_{i}$) to be accessed by any $MP_{i}$. Similarly, each $MP_{i}$ aggregates all the parameter vectors from the neighboring $GP_{j}$, where j≠i. Once this has been achieved, a mixing vector $m_{x}=\left\{w_{i_{i}},w_{i_{2}},\ldots ,w_{i_{k}}\right\}$ is defined for *k* GP as clients for parallel FedAvg learning. The final vector $V_{f}$ is updated as follows [37,38]:

(23) $$\begin{array}{c} V_{f}=\left(m_{x_{1}},m_{x_{2}},\ldots ,m_{x_{k}}\right) \end{array}$$

A mixing matrix $M_{f}$ is defined for the weights obtained from $V_{f}$. This step ensures the diffusion of the individual weights and the secrecy of the individual gradients.

The gradient function for the client batch of $MP_{i}$ is denoted as ∇f(G,ξ), where ξ represents the cardinality N of the client GP batch handled by $MP_{i}$. More intuitively, the gradient function is demonstrated as follows:

(24) $$\begin{array}{c} \nabla f\left(G,\xi \right)=\frac{1}{\xi }\sum_{\forall s\in \xi }\nabla f\left(G,s\right) \end{array}$$

where ∇f(G,s) is the loss gradient of a general sample obtained from any $GP_{i}$. For convergence, we defined an optimal loss function $\nabla^{*}\left(G,\xi \right)$, which is decided by the global server and distributed to ∀MP. Once the data are shared by $GP_{i}$, the aggregator *A* stores the result in the local BC, where the global model picks the gradient.

The algorithm P-FedAvg involves a server and multiple client processes, and every client is associated with a local dataset. The objective was to train the global model and minimize the loss. At any $MP_{i}$, we aggregated the model parameters from its mapped clients while assuring data privacy via a differential privacy mechanism, as outlined in this section. In the server, the initial model parameters are distributed to all clients. Based on a predefined number of rounds, the clients perform local updates on their data using the current model parameters and share their updated parameters with the server. The server aggregates the updated model parameters received from the clients and broadcasts the aggregated parameters to all the clients for the next round of updates. This process continues until convergence is achieved. The client process involves local computation and the sharing of model parameters with the server. Each client computes the gradient of the loss function for its local dataset using the current model parameters and shares the gradient with the server. The server aggregates the clients’ gradients and updates the global model parameters accordingly.

Algorithm 1 shows the details of this process.
**Algorithm 1** The parallel approach to the FedAvg algorithm**Input:** Learning rate η, mixing vector $m_{x}$, $GP_{i}$ initial parameter $G_{i}$, iteration number *N*.
**Output:** Model parameter *X*.
  1:**procedure** Server($x_{0}$)  2:      $G_{mod}\leftarrow x_{0}$  3:      $G_{mod}\leftarrow$*Distribute* ($MP_{1},MP_{2},\ldots ,MP_{q})$  4:      **for** (Each round r=1,2,…,t) **do**  5:            **for** (Every MP=1 to *q*) **do**  6:                 N(c)← *rand_assign*(Cluster_Size)  7:            **end for**  8:            Update $\gamma_{i}\leftarrow \frac{1}{K_{i}}\sum_{i\in K_{i}}G_{i}$  9:            $MP_{i}\leftarrow$*Find_closest_MP* $MP_{j}$10:            A← Exchange model parameters with $MP_{j}$11:            Update ∇f(G,ξ)12:      **end for**13:      **if** ($|\nabla^{*}\left(G,\xi \right)-\nabla f\left(G,\xi \right)\left|\leq 0.01\right)$ **then**14:            Broadcast message "Global Model convergence achieved"15:            Mine block *M* and add to game-chain *C*16:      **else**17:            **REPEAT** steps 4-10 **UNTIL** convergence18:      **end if**19:**end procedure**20:**procedure**CLIENT($x_{0}$)21:      **for** (w←1 to *t*) **do**22:            Compute $\gamma_{w}$23:            Compute local ∇f(x) and share with assigned MP24:            Forward to aggregator *A*25:      **end for**26:      **RETURN** *X*27:**end procedure**


We defined two procedures, *SERVER* and *CLIENT*. On the server side, lines 2–3 show the formation of a parallel $MP_{i}$ from the global model, and lines 4–6 define the random assignment of a GP to any $MP_{i}$. Once the GP is assigned, $\gamma_{i}$ is updated, and the aggregator exchanges model parameters with $MP_{j}$. These steps are shown in lines 7–10. Finally, convergence is achieved and the model loss is minimized between the optimal and current iteration. Lines 12–15 show the conditions. On the other side, the clients compute the local gradient for each iteration and share the local gradient loss with their assigned MP. Lines 18–24 depict these conditions.

To improve the rendering latency of the GPs in the GM environment, we envisioned that our parallel FedAvg algorithm would form a dynamic connected mesh network *M*, where the topology *T* of the node connections (between $MP_{i}$ and $MP_{j}$) is non-variable. A link *l* is established per demand based on the broadcast request defined in Algorithm 1. Further, the 6G-FeMBB service addresses the channel bandwidth to maximize $B_{n}\left(\tau \right)$ and minimize *l*. Thus, the scheme addresses our sub-optimal problems and sets SO(l) to form a unified scheme.

### 3.1. Complexity Analysis

In this subsection, we present an overview of the time complexity of the proposed algorithm. The complexity depends on the number of iterations and the overall computation time per iteration. To analyze the time complexity, we had to consider both the *SERVER* and the *CLIENT* processes. The details are presented below.

#### 3.1.1. Server Process

The complexity of the server process depends on the number of iterations *t* and the computation time required for each iteration. For each iteration, the server performs four functions: cluster assignment, model aggregation, parameter exchange, and gradient computation. The details are presented below.

1. For cluster assignment, we assumed that the dataset size is *d*, with a total of *q* clients. Then, the complexity would be O(d×q).
2. For model aggregation, we used $m_{x}$ as a mixing vector and assumed that the model size is *C*. Thus, for *q* clients, the complexity would be O(q×C).
3. For parameter exchange, we assumed that a ring-based system is preferred to communicate with a neighboring node (neighboring MP), exchanging a message of size *m*. Thus, the communication complexity would be O(t×m) in each iteration. While communicating with the client node, with *t* iterations, the complexity would be O(d×C×t). Thus, the overall complexity would be O(d×q+d×C×t) in the ring-based system.
4. For gradient computation, the server computes the gradient of the objective function using the exchanged parameters. With a dataset size *d* and a model size *C*, the complexity would be O(d×C).

Thus, the overall time-complexity of the server process would be O(d×q+q×C+d×q+d×C×t+d×C).

#### 3.1.2. Client Process

The time complexity of the client process depends on the number of iterations *t* and the computation time per iteration. There are three major tasks at the client node: the local gradient computation, model update, and local model sharing with mapped $MP_{i}$. The details are presented as follows.

1. The time complexity of computing the gradient of the objective function depends on the size of the local dataset and the complexity of the model. Assuming a fixed model architecture, the time complexity of computing the gradient is linear according to the local dataset’s size, denoted by $n_{i}$. Specifically, for each round *w*, the CLIENT function computes the gradient of the objective function for the $i^{th}$ client using its local dataset, which takes $O(n_{i})$ time.
2. The time complexity of updating the model parameters also depends on the size of the local dataset and the complexity of the model. Assuming a fixed model architecture and a fixed number of optimization steps per round, the time complexity of updating the model parameters is linear according to the local dataset’s size, denoted by $n_{i}$. Specifically, for each round *w*, the *CLIENT* function updates its local copy of the model parameters using the computed gradient, which takes $O(n_{i})$ time.
3. The time complexity of sharing the updated model parameters with the server depends on the communication protocol and network bandwidth. Assuming a fixed communication protocol and a fixed number of clients per server, the time complexity of model sharing is proportional to the size of the model parameters, denoted by *b*. Specifically, for each round *w*, the CLIENT function sends its updated model parameters to the assigned MP, which takes O(b) time.

Thus, the overall time complexity of the *CLIENT* node is $O(n_{i}+b)$.

In our proposed scheme, we noticed that the overall *SERVER* node complexity depends on model aggregation, cluster assignment, and fast parameter exchange. In the problem formulation, we wished to maximize $B_{n}\tau$, and thus the model aggregation complexity depends on the amount of individual bandwidth the client node posses. Using a 6G communication service (FeMBB) alleviates this problem. Further, at the server, the size of the local dataset maps onto the problem of maximizing the FL model accuracy $\left(p_{n}^{t}\right)$. The larger the size of the dataset, the more accurately the model can be trained.

On the other hand, the time complexity of the *CLIENT* node is influenced by the number of iterations (t), which is related to the objective of maximizing the accuracy of the FL model $\left(p_{n}^{t}\right)$. The more iterations, the more accurately the model can be trained. The time complexity of the *CLIENT* node is also influenced by the size of the local dataset, which is related to the objective of minimizing the latency of asset transfer. The smaller the dataset size, the faster the asset transfer latency can be minimized.

Thus, the proposed P-FedAvg algorithm with a ring-based communication system between $MP_{i}$s forms an effective load-balancing mechanism and was thus deemed conducive to a higher accuracy and faster convergence in FL tasks compared to other methods such as centralized FL and non-ring-based parallel FL algorithms. Additionally, our method can handle heterogeneous client environments with varying computation capabilities and communication bandwidths, a common real-world scenario. Therefore, our method is superior in scalability, adaptability, and performance.

## 4. Performance Evaluation

This section presents the performance evaluation of the *Game-o-Meta* scheme based on some defined parameters, namely, the mining cost of transactional updates by $GP_{n}$, the GO (avatar) rendering time on the 6G channel, the energy consumption of the FL dataset setup, the federated RL average reward plot, the overall model accuracy, and a comparative analysis of different FL aggregation algorithms. After the simulation results, we present the formal security analysis of the proposed scheme.

### 4.1. Experimental Setup

For the simulation of the BC node, we used the Ethereum Remix virtual machine 1.3.6 native IDE. This system included an Intel i5 processor with 8 GB RAM, 128 GB SSD, and Ubuntu 80.04 LTS installed. For FL learning, we trained the machine learning model using MNIST with skewed data [39]. Each device from a federated group of 100 mobile game samples was randomly selected from the training dataset. We varied the number of GPs to 60 for a federated environment, resulting in a training dataset of up to 6000 combinations. We used three different neural networks to train the model with 0, 64, and 512 hidden layers of neurons. We trained the model with a different dataset and different Earth mover’s distance settings, and, to measure the energy consumed in training, we used Raspberry Pi. The details of the simulation parameters for the experimental setup are presented in Table 2.

### 4.2. Simulation Results

**Figure 4:**
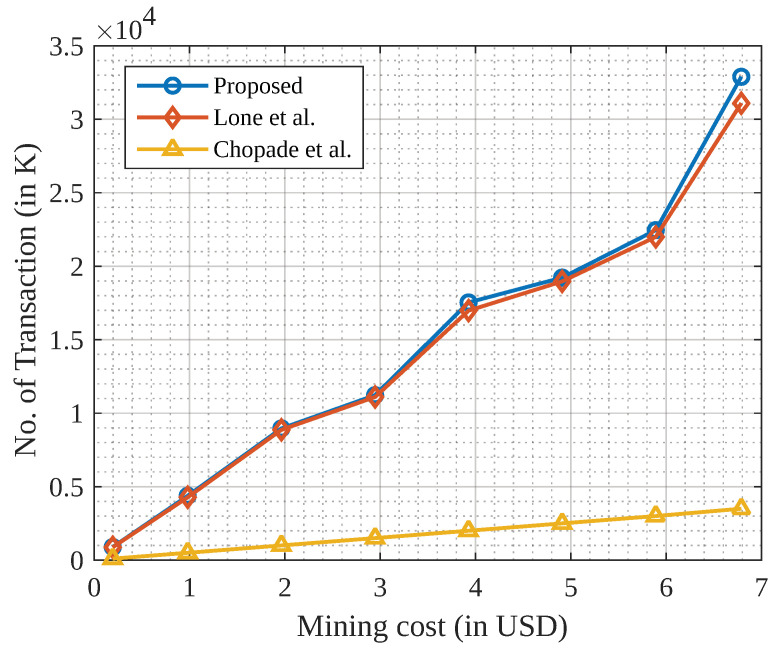
Mining cost of transactions stored in BC.

Here, we present the simulation results of the proposed scheme. Figure 4 represents the mining cost of storing the weights ($W_{GP_{n}}$) in the BC via the IPFS.

First, the $W_{GP_{n}}$ is stored in the IPFS, and the IPFS generates a 32-byte content address that is stored in a block of the BC. For 1000 gradient updates, the approximate size of the block would be 3.8 kilobytes (KB). With this block size, the cost of storing based on the mining incentive and the current rate of Ethereum 2023 (Q1) is USD 14.82 per KB. Based on the above computation, we compared the mining cost of storing data on the BC against related schemes [40,41] and demonstrated a significant improvement of 78.24% in the mining cost.

**Figure 5:**
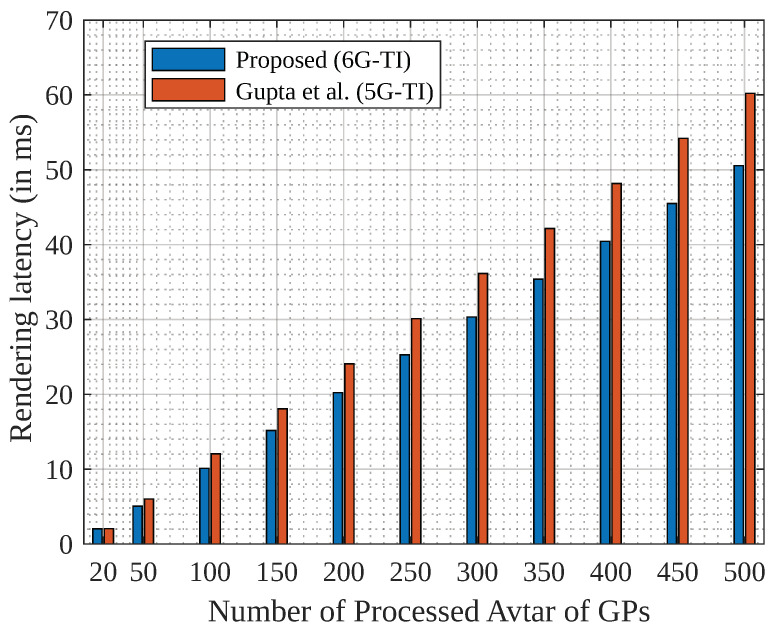
Rendering latency of GP avatars.

Figure 5 In terms of metaverse rendering, we considered the 6G-TI channel, with the FeMBB service.

A realistic scenario consists of a 4K scene of about 8294400 pixels with a 12-bit color depth and a 120-frames-per-second frame rate. The transmission time of the image in 5G-TI and 6G-TI would be 111 ms and 101 ms, respectively, and the 3D rendering avatar would require a transmission of 4.32 Tbps [42]. Thus, the rendering time for 5G-TI and 6G-TI was found to be 4.9 ms and 0.049 ms, which resulted in the overall latency of 5G-TI being ≈ 115 ms and that of 6G-TI being ≈ 101 ms [43,44]. The figure shows an improvement of ≈36% compared to traditional 5G-TI communication.

**Figure 6:**
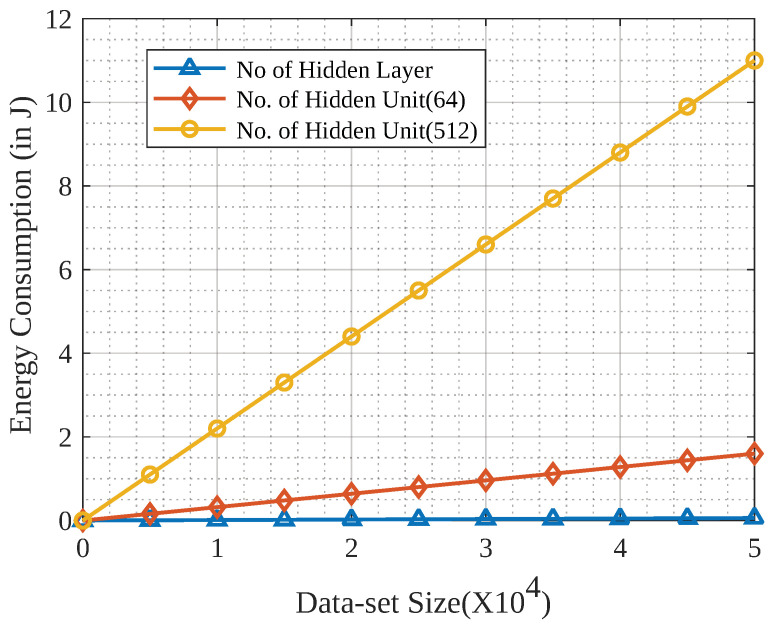
Measurement of device energy consumption.

Figure 6 shows the energy consumption of training the global model based on the $W_{GP}$ received by the gradients from all GPs.

The energy demand of an ML model can be defined as the amount of energy required to train the model on a given hardware platform, which is a critical factor in designing and deploying ML models, as it directly affects the cost, efficiency, and environmental impact of these systems. One way to mathematically represent the energy demand of an ML model is to consider the number of operations required for training or inference. Let us assume that we have a neural network model with *L* layers, and each layer *l* has $n_{l}$ neurons. The number of operations required for training the model on a dataset of size N can be approximated as follows:

(25) $$\begin{array}{c} E=\sum_{i=1}^{n}N_{i}\cdot f_{i}\cdot V_{dd}^{2}\cdot C_{i} \end{array}$$

where *E* is the total energy demand of the ML model, *n* is the total number of layers in the model, $N_{i}$ is the number of neurons in layer i, $f_{i}$ is the average spiking frequency of neurons in layer i, $V_{dd}$ is the supply voltage of the neurons, and $C_{i}$ is the total capacitance of the neurons in layer i. Here, the term $n_{l}^{2}$ represents the number of operations required to compute the inner product of the weights and activations of a single neuron in layer *l*. The sum of all layers accounts for the total number of neurons in the model. Changing the number of hidden layer units also affects the model’s accuracy. The number of hidden layer units is a hyperparameter that needs to be tuned to optimize the performance of a neural network. Increasing the number of hidden layer units can allow the network to learn more complex relationships between the input and output, potentially leading to higher accuracy. However, increasing the number of hidden layer units also increases the computational complexity of the network, which can lead to higher energy consumption. Thus, we adjusted the number of hidden layer units in the simulations to find a balance between accuracy and energy demand. We considered the hidden layer units to determine the optimal configuration for the proposed scheme. As we observed, the training duration for local GPs was linear, and as we increased the dataset size, the time required to train the model grew simultaneously. The neural network model was trained with 0, 64, and 512 hidden neural network layers. From the figure, one can observe that as the complexity of the training increased, the energy required for the model’s training also increased.

**Figure 7:**
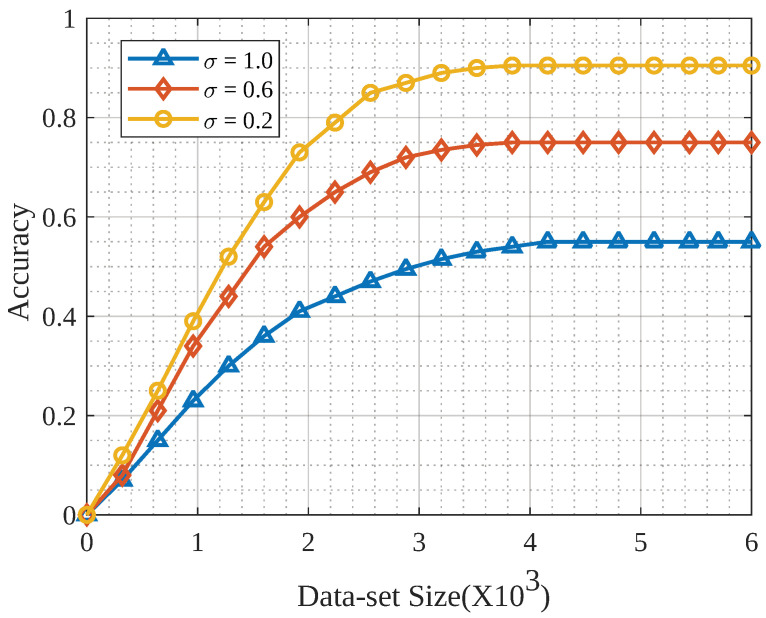
Model accuracy for different dataset sizes.

Figure 7 presents the model accuracy with a varying dataset size.

To simulate this relationship, we considered the Earth mover’s distance (EMD) metric, which signifies the dissimilarity between two probability distributions. In FL, EMD is used to measure the distance between the local models of clients and the global model. The EMD impacts the performance of FL algorithms, as it affects how the local models of the clients are combined to form the global model. A smaller EMD indicates that the clients’ local models are more similar and hence can be combined more easily to form the global model. A larger EMD indicates that the local models are more dissimilar; hence, it may be more difficult to combine them to form the global model. Therefore, a lower EMD is generally preferred for FL.

When the dataset size was small, each client had fewer samples to train the model, which meant that the distribution of model parameters across clients was more similar. This was because, with fewer samples, there was less room for variation in the learned model parameters.

Therefore, it was expected that when the dataset size was small, the EMD value would be lower compared to when the dataset size was large and each client had more samples and, therefore, there was more variability in the learned model parameters. The results were consistent with the above explanation. We used different Earth mover’s distances (σ) to check the model’s accuracy and evaluate the dissimilarity between the two multidimensional distributions of data points. The position and points were the points in N-dimensional space that were critical. When the size of the dataset was relatively small, the accuracy of the model increased dramatically, converging to a certain level when the size was large.

**Figure 8:**
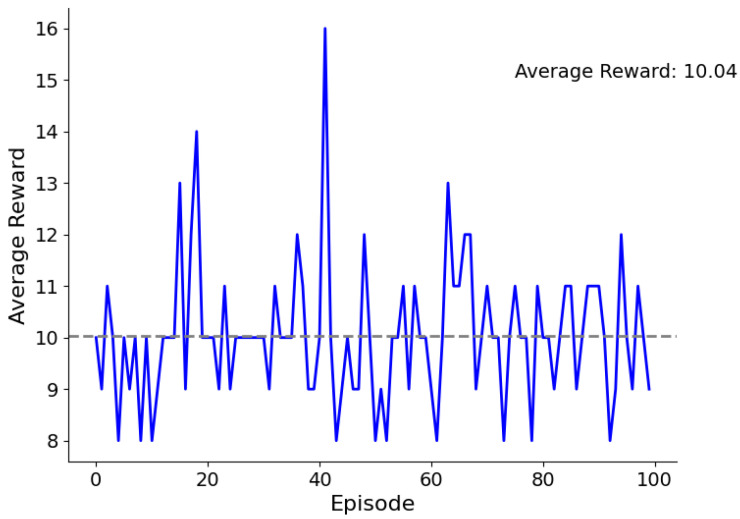
RL average rewards plot.

To assess the performance of our federated RL algorithm, we considered a plot of the average reward obtained during the training of the RL algorithm. We considered the number of episodes and the average reward per episode. The initial RL parameters were set as depicted in Table 1. Figure 8 presents the plot details.

As one can see from the plot, the average reward was low at first and gradually increased as the number of episodes increased. This indicated that the RL algorithm was learning and improving over time. The Q-learning algorithm uses a Q-value function to estimate the expected rewards of a particular action in a given state. The algorithm iteratively updates the Q-values based on the observed rewards and chooses the action with the highest Q-value in each state.

The plot shows the average reward obtained per episode over 100 episodes of training. The blue line represents the rewards obtained in each episode, and the gray dashed line represents the average reward over all episodes. The plot indicates that the Q-learning algorithm improved the game player experience by gradually increasing the average reward over the training episodes. Thus, the algorithm performance improved as it learnt to optimize the action selection process in the GM environment. The reward obtained per episode increased as the algorithm became better at selecting actions.

In an FL environment, client devices are heterogeneous, with limited resources, and each device has capabilities such as computing power and network bandwidth. Normally, a client randomly decides to participate in the training process, which affects the training and unbalances the model accuracy. Therefore, it was important to ensure that the clients participated in the training process. The FedAvg approach randomly selected a client for participation; the model performed poorly when the data were highly dependent and not distributed identically.

**Figure 9:**
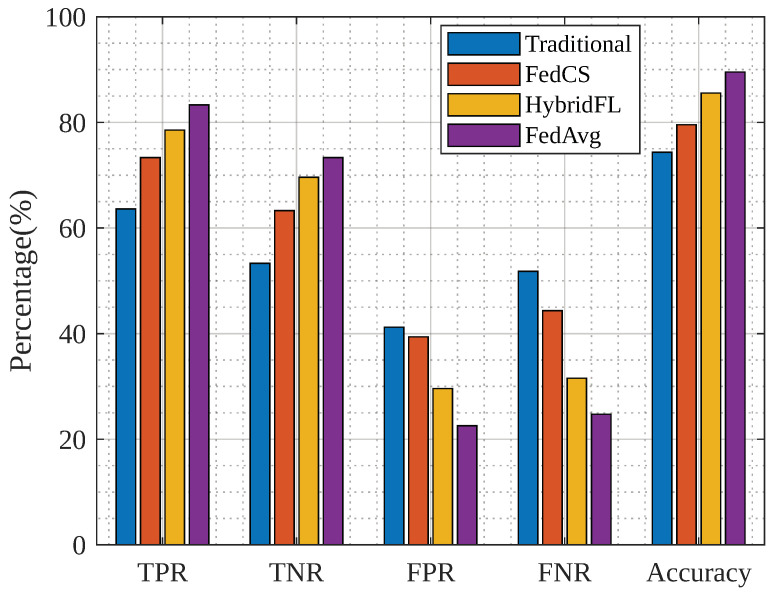
Comparative analysis of different aggregation algorithms.

Figure 9 represents a comparison of the traditional, FedCS [45], and HybridFL [46] aggregation algorithms with FedAvg.

We considered the true-positive rate (TPR), true-negative rate (TNR), false-positive rate (FPR), false-negative rate (FNR), and accuracy as the parameters for algorithm selection. In the figure, TPR is compared against the traditional approaches for classification, TNR for categorization, FPR for classification, and FNR for classification, as well as classification accuracy. We used the FedAvg algorithm to achieve better accuracy based on these parameters.

### 4.3. Formal Security Analysis

**Theorem** **1.** *The proposed P-FedAvg scheme is secure against passive attacks, wherein an adversary tries to obtain sensitive information from the encrypted gradients or weights. This is achieved by using homomorphic encryption for gradient exchange and aggregation.*

**Proof.**  Let $g_{n}^{t}$ denote the gradient computed on the *n*-th peer device at iteration *t*. The gradient is encrypted before transmission to the GS using a public key encryption scheme, as follows:

(26) $$\begin{array}{c} {\hat{g}}_{n}^{t}=E_{PK}\left(g_{n}^{t}\right) \end{array}$$

where $E_{PK}$ is the encryption function using the public key PK. Assuming the adversary intercepts the encrypted gradient ${\hat{g}}_{n}^{t}$, the adversary would not be able to obtain the plaintext gradient $g_{n}^{t}$ without the private key $SK_{n}$. This is due to homomorphic encryption, which allows operations on ciphertexts to be performed as if they are being performed on plaintexts. It also ensures that the plaintext gradient is not revealed during aggregation. Hence, the proposed P-FedAvg scheme is secure against passive attacks. □

**Theorem** **2.** *The proposed P-FedAvg scheme is secure against active attacks launched by malicious GSs.*

**Proof.**  Let $g_{n}^{t}$ be the gradient computed on the *n*-th peer device at iteration *t*. Let $E_{PK}$ be the encryption function using the public key PK, and $D_{SK_{n}}$ the decryption function using the private key $SK_{n}$ of the *n*-th peer device. Let $g^{t}$ be the aggregated gradient at iteration *t*, computed as the sum of the encrypted gradients, as follows:

(27) $$\begin{array}{c} g^{t}=\sum_{n\in N}{\hat{g}}_{n}^{t} \end{array}$$

where ${\hat{g}}_{n}^{t}=E_{PK}\left(g_{n}^{t}\right)$. Suppose a malicious GS launches an active attack to modify the aggregated gradient $g^{t}$ by adding a malicious gradient $\Delta g^{t}$ as follows.

(28) $$\begin{array}{c} g^{\prime t}=g^{t}+\Delta g^{t} \end{array}$$

where $g^{\prime t}$ is the modified gradient. The game server then generates a new set of global weights $w^{t+1}$ by performing a gradient descent step using the modified gradient, as follows:

(29) $$\begin{array}{c} w^{t+1}=w^{t}-\alpha \nabla g^{\prime t} \end{array}$$

However, the malicious gradient $\Delta g^{t}$ is encrypted using the public key PK, and thus the GS cannot directly modify it. Instead, the GS would have to compute the encrypted version of the malicious gradient as follows:

(30) $$\begin{array}{c} {\hat{\Delta g}}^{t}=E_{PK}\left(\Delta g^{t}\right) \end{array}$$

and add it to the encrypted gradients received from the peer devices. This step is shown below.

(31) $$\begin{array}{c} {\hat{g^{\prime }}}^{t}=\sum_{n\in N}\left({\hat{g}}_{n}^{t}+{\hat{\Delta g}}^{t}\right) \end{array}$$

However, since homomorphic encryption is used, the GS cannot perform addition on the encrypted gradients without knowing the private keys $SK_{n}$ of the peer devices. Thus, a malicious attack is not feasible. Therefore, the proposed P-FedAvg scheme is secure against active attacks launched by malicious GSs. □

**Theorem** **3.** *The proposed P-FedAvg scheme is secure against compromised peer attacks.*

**Proof.**  Let $g^{t}$ be the aggregated gradient at iteration *t*, computed as the sum of the encrypted gradients, as follows:

(32) $$\begin{array}{c} \hat{g_{n}^{t}}=\sum_{n\in N}\hat{g_{n}^{t}} \end{array}$$

where $\hat{g_{n}^{t}}=E_{PK}\left(g_{n}^{t}\right)$. Suppose a compromised peer attack is launched, where a peer device with private key $SK_{c}$ is compromised by an attacker. The attacker can use $SK_{c}$ to decrypt the encrypted gradient $\hat{g_{n}^{t}}$ and obtain the plaintext gradient $g_{c}^{t}$. The attacker can then modify the plaintext gradient and encrypt it using the public key PK, generating a new encrypted gradient $\hat{g{{}^{\prime }}_{c}^{t}}$, as follows:

(33) $$\begin{array}{c} \hat{g{{}^{\prime }}_{c}^{t}}=EPK\left(g{{}^{\prime }}_{c}^{t}\right) \end{array}$$

where $\hat{g{{}^{\prime }}_{c}^{t}}$ is the modified plaintext gradient. However, since the other peer devices are still computing their gradients, the attacker cannot modify their encrypted gradients without knowing their private keys $SK_{n}$. Thus, the attacker cannot modify the aggregated gradient $g^{t}$. Therefore, the proposed P-FedAvg scheme is secure against compromised peer attacks. □

## 5. Conclusions and Future Scope

In the future, the gaming industry will involve immersive, realistic GE experiences for GPs, and GMs will soon become the norm. AI-enabled GMs could leverage XR engines to enable smooth interactions and improve the QoI for GPs. In this paper, we proposed a scheme, *Game-o-Meta*, that addresses the requirements of secured and personalized GEs for GPs, with GE data being trained on local devices via FL. We envisioned a 6G-TI channel to allow real-time haptics control and support large numbers of concurrent GP connections over the metaverse environment. FL also assures the privacy of GP data, as they are not shared centrally. A FedAvg algorithm was presented, wherein the communication costs were minimized due to the 6G-P2P channel. Further, the scheme considered transactional payments and asset transfers via BC and NFTs. To address the scalability concerns of BC, we determined that local GP data would be stored in local offline IPFSs and accessed through a content key shared with any authorized user. Thus, the presented scheme formed an umbrella approach for P2P GM networks (in terms of effective learning, communication delay, and secured transactions).

In the future, the authors will extend this article by achieving weight FL aggregation over a multi-GP scenario via the secured multi-part computation algorithm, with the collaborating GPs keeping the game inputs private and jointly computing a function to obtain the gradient inputs.

## Figures and Tables

**Table 1:** Comparative analysis of proposed scheme and SOTA approaches.

Author	Year	Objectives	Application	Limitations
Proposed herein	2023	A parallel FL-based averaging (P-FedAvg) scheme for GM wherein gradient exchange is managed via BC	Gaming metaverses	An evaluation of the privacy-preserving scheme was not carried out
Zelenyanszki et al. [27]	2023	The authors suggested creating a framework for promoting privacy awareness using ML algorithms to recognize harmful patterns and send notifications to users	Metaverses and digital transformation	Privacy preservation and the identification of a malicious users were not considered
Zhou et al. [28]	2023	This paper presented a metaverse-based MAR system called NOMA-FL	Metaverses and augmented reality	Communication delays and global model convergence were not considered
Hui et al. [29]	2022	A model for hierarchical FL in the gaming industry that safeguarded user privacy and leveraged mobile edge computing	Gaming, autonomous driving, and health monitoring	The model convergence was not fast
Zhang et al. [30]	2022	A differential privacy FL model with client incentives for training	The identification of malicious participants	Security and privacy concerns were not addressed
Gupta et al. [31]	2022	To devise a model called FL GAMES for learning and observing causal features	Gaming	The time complexity was high, and the speed of the communication rounds was low
Lee et al. [32]	2021	To utilize asynchronous FL (AFL) in the wireless distributed learning network field for metaverse implementations	The technology of autonomous twins for metaverses	The wireless network radio resources are restricted, and posterior distribution is difficult
Fan et al. [33]	2021	To implement a blockchain-based FL system and propose an incentive mechanism for creating a transparent trading platform	Word prediction	Higher computational cost and delay
Jiang et al. [34]	2021	To implement a mobile crowd FL gaming system consisting of a collection of mobile devices and a central server	Gaming environment	The unpredictable upload time resulting from the mobile devices led to increased time for local training
Yu et al. [35]	2020	FL incentivization for data owners was implemented in the waiting time calculations with payoffs from the received information	The training of FL data	Privacy and quality improvements were required

**Table 2:** Simulation parameters.

Parameter	Value
# of $MP_{i}$s	6
# of GPs per MP	10
Epochs	1000
Dataset size	6000
Training samples	5000
Testing samples	1000
Batch size	100
Optimizer	SGD
RL episodes	100
Max time steps/episode	1000
Learning rate (α)	0.01
Discount factor (γ)	0.99
Initial value function	0
Initial Q-value	0

## Data Availability

No data are associated with this research work.
